# The role of recipient myosteatosis in graft and patient survival after deceased donor liver transplantation

**DOI:** 10.1002/jcsm.12669

**Published:** 2021-02-01

**Authors:** Zoltan Czigany, Wiebke Kramp, Isabella Lurje, Hannah Miller, Jan Bednarsch, Sven Arke Lang, Tom Florian Ulmer, Philipp Bruners, Pavel Strnad, Christian Trautwein, Martin Wolfgang von Websky, Frank Tacke, Ulf Peter Neumann, Georg Lurje

**Affiliations:** ^1^ Department of Surgery and Transplantation University Hospital RWTH Aachen Aachen Germany; ^2^ Institute of Radiology University Hospital RWTH Aachen Aachen Germany; ^3^ Department of Internal Medicine III University Hospital RWTH Aachen Aachen Germany; ^4^ Department of Surgery University Hospital Bonn Bonn Germany; ^5^ Department of Hepatology and Gastroenterology, Campus Charité Mitte Campus Virchow‐Klinikum Charité—Universitätsmedizin Berlin Berlin Germany; ^6^ Department of Surgery, Campus Charité Mitte Campus Virchow‐Klinikum Charité—Universitätsmedizin Berlin Berlin Germany; ^7^ Department of Surgery Maastricht University Medical Centre (MUMC) Maastricht The Netherlands

**Keywords:** Liver transplantation, Body composition, Myosteatosis, Sarcopenia, Graft survival, Patient survival

## Abstract

**Background:**

Myosteatosis is associated with perioperative outcomes in orthotopic liver transplantation (OLT). Here, we investigated the effects of body composition and myosteatosis on long‐term graft and patient survival following OLT.

**Methods:**

Clinical data from 225 consecutive OLT recipients from a prospective database were retrospectively analysed (May 2010 to December 2017). Computed tomography‐based lumbar skeletal muscle index (SMI) (muscle mass) and mean skeletal muscle radiation attenuation (SM‐RA) (myosteatosis) were calculated using a segmentation tool (3D Slicer). Patients with low skeletal muscle mass (low SMI) and myosteatosis (low SM‐RA) were identified using predefined and validated cut‐off values.

**Results:**

The mean donor and recipient age was 55 ± 16 and 54 ± 12 years, respectively. Some 67% of the recipients were male. The probability of graft and patient survival was significantly lower in patients with myosteatosis compared with patients with higher SM‐RA values (*P* = 0.011 and *P* = 0.001, respectively). Low skeletal muscle mass alone was not associated with graft and patient survival (*P* = 0.273 and *P* = 0.278, respectively). Dividing the cohort into quartiles, based on the values of SMI and SM‐RA, resulted in significant differences in patient but not in graft survival (*P* = 0.011). Even though multivariable analysis identified low SM‐RA as an important prognostic marker (hazard ratio: 2.260, 95% confidence interval: 1.177–4.340, *P* = 0.014), myosteatosis lost its significance when early mortality (90 days) was excluded from the final multivariable model. Patients with myosteatosis showed significantly higher all‐cause mortality and in particular higher rates of deaths due to respiratory and septic complication (*P* = 0.002, *P* = 0.022, and *P* = 0.049, respectively).

**Conclusions:**

Preoperative myosteatosis may be an important prognostic marker in patients undergoing deceased donor liver transplantation. The prognostic value of myosteatosis seems to be particularly important in the early post‐operative phase. Validation in prospective clinical trials is warranted.

## Introduction

Pathological variations of body composition (BC) are frequently seen in critical illness and have been associated with inferior clinical outcomes in a variety of medical conditions.^1^, ^2^ Accordingly, malnutrition and muscle wasting are characteristic for patients with chronic liver disease,^1^, ^3^ and as such, nutritional screening as well as the assessment of BC were recently implemented in the European Association for the Study of the Liver and American Association for the Study of Liver Diseases practice guidelines on nutrition in chronic liver disease.^4^, ^5^

Even though numerous methods are used for clinical BC assessment,^6^ the direct quantification of muscle and fat tissue using cross‐sectional computed tomography (CT) imaging is considered the gold standard in transplant waiting list candidates and in patients with chronic liver disease.^1^, ^4^, ^6^ While sarcopenia—the pure loss of muscle mass and strength—was linked to clinical outcomes in various diseases,^3^, ^6^, ^7^, ^8^, ^9^, ^10^ the long‐term prognostic value of muscle quality (muscle density or myosteatosis) compared with muscle quantity (muscle mass or sarcopenia) remains to be determined.^1^, ^3^, ^7^, ^8^, ^9^

Nowadays, an increasing number of extended criteria donor allografts are utilized that were previously considered unsuitable for transplantation.^11^, ^12^ Because of that, a careful selection and matching of donors and recipients are essential to improve allograft utilization and post‐orthotopic liver transplantation (OLT) outcomes.^13^ Recent studies by our group and others demonstrated a high prevalence of BC alterations in patients with end‐stage liver disease. In particular, myosteatosis was identified as an important prognostic factor in predicting adverse perioperative outcomes in patients undergoing OLT.^9^, ^10^, ^14^, ^15^, ^16^, ^17^

Because myosteatosis seems to be an accurate predictor for clinical outcomes, the goal of the present study was to assess the performance of CT‐based recipient BC profiling in predicting long‐term graft and patient survival in individuals undergoing deceased donor OLT.

## Methods

### Patients and ethics

Between May 2010 and December 2017, all consecutive patients undergoing OLT at the University Hospital RWTH Aachen (UH‐RWTH), Aachen, Germany, were considered for inclusion. Exclusion criteria were defined as (i) CT scans older than 6 months and/or those not including images from the third lumbar vertebra (L3) level^14^ and (ii) living related or split liver transplantation. Patients undergoing re‐OLT have been assessed only for the primary transplantation, and consecutive transplantations were included in the follow‐up. The study was conducted at the UH‐RWTH in accordance with the current version of the Declaration of Helsinki as well as the Declaration of Istanbul and the International Conference on Harmonisation Good Clinical Practice guidelines. The study was approved by the responsible Institutional Review Board of the RWTH Aachen University (EK 047/18). Informed consent was waived because of the retrospective study design and collection of readily available clinical data.

### Segmentation and body composition analysis

All CT scans were performed by using a state‐of‐the‐art multi‐slice CT scanner. The technical parameters for CT imaging were described before.^14^

**Figure 1:**
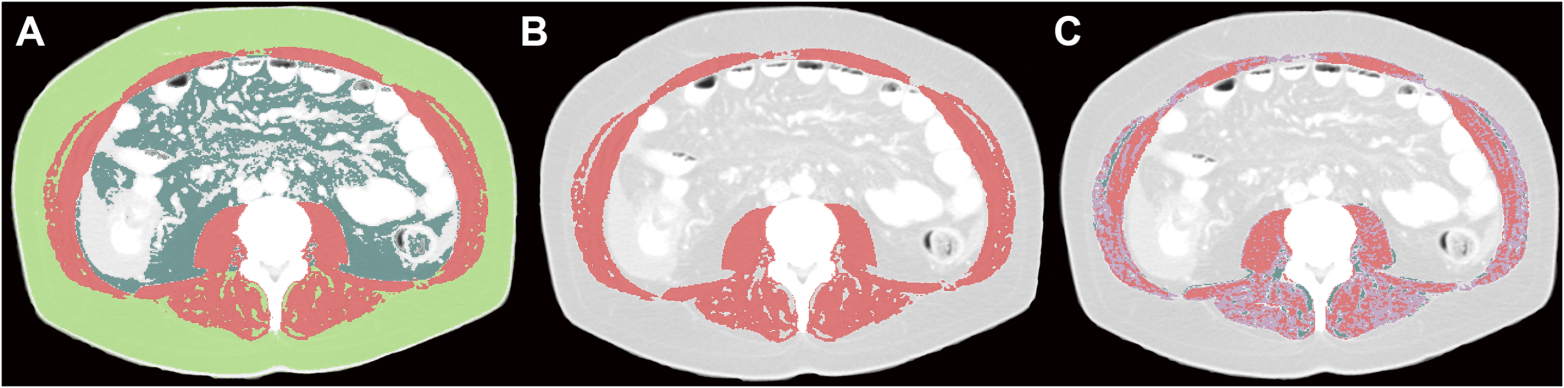
Segmentation of cross‐sectional computed tomography images at the level of the third lumbar vertebra. Representative axial images of the preoperative CT scan of a 55‐year‐old male patient underwent liver transplantation for hepatocellular carcinoma during the study period. (A, B) Skeletal muscle area (red) was determined by using computed tomography attenuation values of −29 to 150 HU. Subcutaneous fat area (light green) was defined as attenuation values of −190 to −30 HU. For visceral fat area (dark green), −150 to −50 HU attenuation values were used. In this patient with considerable structural alterations of the skeletal muscle, panel C shows the amount of intramuscular adipose tissue in dark green (−190 to −50 HU). While normal attenuation muscle has been marked red (+30 to 150 HU), myosteatotic, low attenuation muscle was delineated in violet (−29 to 29 HU). Note the large amount of low attenuation muscle (violet colour) in panel C, indicating the presence of low‐quality myosteatotic muscle.

Data of the most recent preoperative CT imaging were retrieved from digital storage in the picture archiving and communication system. Body composition analysis was performed as previously described by our group.^14^ Briefly, a single cross‐sectional CT image at the level of L3 was used, and the segmentation of skeletal muscle and adipose tissue was performed using the 3D Slicer software platform Version 4.1 and BC module (https://www.slicer.org/ ^18^) in a semi‐automatic fashion. Skeletal muscle area was identified and quantified by using attenuation values of −29 to 150 Hounsfield units (HU). Skeletal muscle index [SMI; the indicator of low skeletal muscle mass (SMM) or structural aspect of sarcopenia] has been calculated by normalizing the measured muscle area to the square height of the patient (cm^2^/m^2^).^14^ Skeletal muscle radiation attenuation (SM‐RA) as an indicator of muscle density and myosteatosis has been recorded in HU.^14^ Visceral fat was quantified by using the attenuation values −150 to −50 HU. To identify subcutaneous adipose tissue, the attenuation values of −190 to −30 were used (*Figure* 1).^14^ All measurements were performed by the same researcher, experienced in complex BC analyses.

Based on the SMI and SM‐RA, reduced SMM and myosteatosis were defined using cut‐off values determined specifically for patients on OLT waiting list (SMI: female 39 cm^2^/m^2^ and male 50 cm^2^/m^2^; SM‐RA < 41 HU for patients with a body mass index up to 24.9 kg/m^2^ and <33 HU for patients with a body mass index ≥25 kg/m^2^).^6^, ^14^, ^19^ Based on previous literature findings from patient cohorts with terminal liver disease, no adjustment for sex was made for SM‐RA.^6^, ^14^

### Perioperative management and data collection

All OLT waiting list indications were discussed and decided within a multidisciplinary liver transplantation board meeting in accordance to the German national and Eurotransplant guidelines (Eurotransplant Manual Version 5.5, Eurotransplant Foundation, Leiden, the Netherlands). Organ allocation followed national and Eurotransplant regulations. The liver transplantation procedure was performed using a standardized approach of total cava replacement as previously described.^14^, ^20^ The standard perioperative care and immunosuppression regimen consisted of basiliximab, tacrolimus, mycophenolate mofetil, and corticosteroids.^14^, ^20^

Clinical data were obtained from a prospective institutional database and analysed, retrospectively. Various OLT risk scores (*Table* 1) have been calculated as described before.^14^, ^23^, ^29^, ^30^ Extended criteria donor allografts were defined according to the definitions of the German Medical Chamber.^22^ To assess post‐transplant early allograft dysfunction, the Olthoff criteria were adopted.^27^

**Table 1:** Donor and recipient characteristics

Characteristics	All patients (*n* = 225)	Patients without 90 day mortality (*n* = 206)
Donor age (years)	55 ± 16	55 ± 16
Donor BMI	30 ± 8	30 ± 8
Donor sex ratio (F:M)	108 (48%):117 (52%)	100 (48%):106 (52%)
DRI^a^	1.77 ± 0.35	1.76 ± 0.35
Donor cause of death	CVA 138 (61%)	CVA 130 (63%)
	Anoxia 51 (23%)	Anoxia 47 (23%)
	Trauma 25 (11%)	Trauma 21 (10%)
	Other 11 (5%)	Other 8 (4%)
ECD^b^	154 (68%)	139 (68%)
Recipient age (years)	54 ± 12	54 ± 12
Recipient BMI	27 ± 5	27 ± 5
Recipient sex ratio (F:M)	75 (33%):150 (67%)	66 (32%):140 (68%)
Aetiology of liver disease	ALF 31 (14%)	ALF 29 (14%)
	HCC 63 (28%)	HCC 57 (28%)
	Alc. cirrhosis 45 (20%)	Alc. cirrhosis 40 (19%)
	Viral 15 (7%)	Viral 13 (6%)
	PSC/PBC 21 (9%)	PSC/PBC 20 (10%)
	Graft failure 4 (2%)	Graft failure 3 (2%)
	Other 46 (20%)	Other 44 (21%)
Pre‐transplant Child–Pugh score	7 ± 2	7 ± 2
Pre‐transplant labMELD	20 ± 11	19 ± 10
BAR score^c^	9 ± 6	8 ± 5
SOFT score^d^	15 ± 10	14 ± 9
Recipient pre‐transplant ICU	56 (25%)	45 (22%)
Recipient pre‐transplant abdominal surgery	82 (36%)	72 (35%)
Recipient pre‐transplant encephalopathy	90 (40%)	82 (40%)
Karnofsky performance score^e^	60 ± 25	62 ± 24
Cold ischaemic time (min)	516 ± 139	511 ± 134
Warm ischaemic time (min)	46 ± 7	46 ± 7
Intraoperative red blood cell transfusions (units)	9 ± 8	9 ± 8
Intraoperative fresh frozen plasma transfusions (units)	18 ± 10	17 ± 10
Post‐operative red blood cell transfusions (units)^f^	4 ± 6	3 ± 5
Post‐operative fresh frozen plasma transfusions (units)^f^	6 ± 11	5 ± 8
90 day ≥CD3b complications^g^	114 (51%)	95 (46%)
90 day mortality	19 (8%)	Not applicable
Early allograft dysfunction *n* (%)^h^	60 (27%)	53 (26%)
ICU stay (days)	14 ± 23	12 ± 21
Hospital stay (days)	43 ± 42	42 ± 43
90 day CCI^i^	54 ± 33	50 ± 31

ALF, acute liver failure; BAR, balance of risk; BMI, body mass index; CCI, comprehensive complication index; CD, Clavien–Dindo classification; CVA, cerebrovascular accident; DRI, donor risk index; ECD, extended criteria donor allografts; HCC, hepatocellular carcinoma; ICU, intensive care unit; MELD, model for end‐stage liver disease; PBC, primary biliary cholangitis; PSC, primary sclerosing cholangitis; SOFT, survival outcomes following liver transplantation.

Values were given as mean ± standard deviation or *n* (%).

^a^ Refers to Feng *et al*.^21^

^b^ Refers to German Medical Chamber guidelines.^22^

^c^ Refers to Schlegel et al.^23^

^d^ Refers to Rana et al.^24^

^e^ Refers to Kelly et al.^25^

^f^ Refers to blood products given during the first 7 days after orthotopic liver transplantation

^g^ Refers to Clavien et al.^26^

^h^ Refers to Olthoff et al.^27^

^i^ Refers to Slankamenac et al.^28^

Post‐operative morbidity was evaluated for all surgical complications observed during the first 90 days after OLT according to the Clavien–Dindo classification and quantified using the comprehensive complication index.^26^, ^28^ Recipient pre‐OLT performance status has been assessed using the Karnofsky performance score (KPS).^25^ Post‐operative transfusions were defined as any blood products given within the first 7 days following OLT. Blood products administered later in the post‐operative period were categorized as post‐operative complications according to the recommendations of the Clavien–Dindo classification.^26^ Length of intensive care unit (ICU) stay represents the initial stay after the OLT procedure until the transfer of the patient to our standard care transplantation unit. Hospital stay was defined by the date of admission for OLT and the day of discharge from the UH‐RWTH. Readmission to the ICU was included in the total hospital stay. Our transplantation outpatient department as well as the responsible general practitioner and/or hepatologist provided all follow‐up data used for the survival analyses in this study.

### Study endpoints and statistical analysis

Probability of patient survival at 5 years was chosen as the primary endpoint for the survival analyses. Five year graft survival was used as secondary endpoint.

Categorical data are presented in the form of numbers and percentages. Data derived from continuous variables were presented as mean and standard deviation. Categorical data were compared using the *χ* ^2^ test or Fisher's exact test according to scale and number counts. The associations of graft and patient survival with BC characteristics were assessed using univariate and multivariable Cox proportional hazards regression models. Survival curves were generated by the Kaplan–Meier method and compared with the log‐rank test. All *P*‐values <0.05 were considered statistically significant. Statistical analysis has been performed using SPSS Statistics v24 (IBM Corp., Armonk, NY, USA).

## Results

### Patient and graft characteristics

Out of all 357 consecutive OLTs performed, 225 patients met the predefined inclusion and exclusion criteria.^14^ Among 132 excluded patients were recipients of living related (*n* = 5) or split liver allografts (*n* = 4), and cases without sufficient preoperative CT imaging (*n* = 123). Patients' characteristics and perioperative outcome data of the cohort were in part reported previously^14^ and are also summarized in *Table* 1.

**Figure 2:**
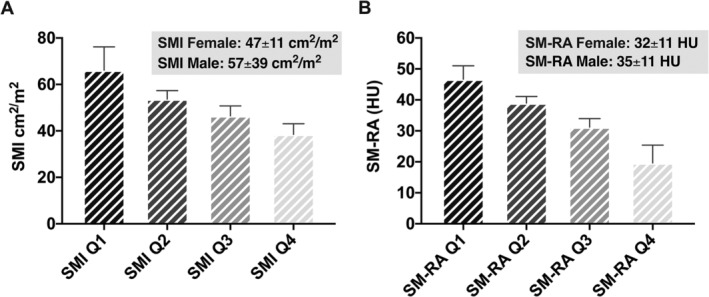
Distribution of skeletal muscle index (SMI) and skeletal muscle radiation attenuation (SM‐RA) values within the patient cohort. (A) Quartile‐based distribution of lumbar 3 SMI. (B) Quartile‐based distribution of lumbar 3 SM‐RA.

The median time between the CT imaging used for segmentation and OLT was 5 weeks (range 0–24). The mean SMI was 57 ± 39 cm^2^/m^2^ for male patients and 47 ± 11 cm^2^/m^2^ for female patients. The mean SM‐RA was 35 ± 11 HU for male patients and 32 ± 11 HU for female patients, respectively. *Figure* 2 depicts the distribution of the SMI and SM‐RA values within the patient cohort.

### Impact of low muscle mass and myosteatosis on long‐term graft and patient survival

**Figure 3:**
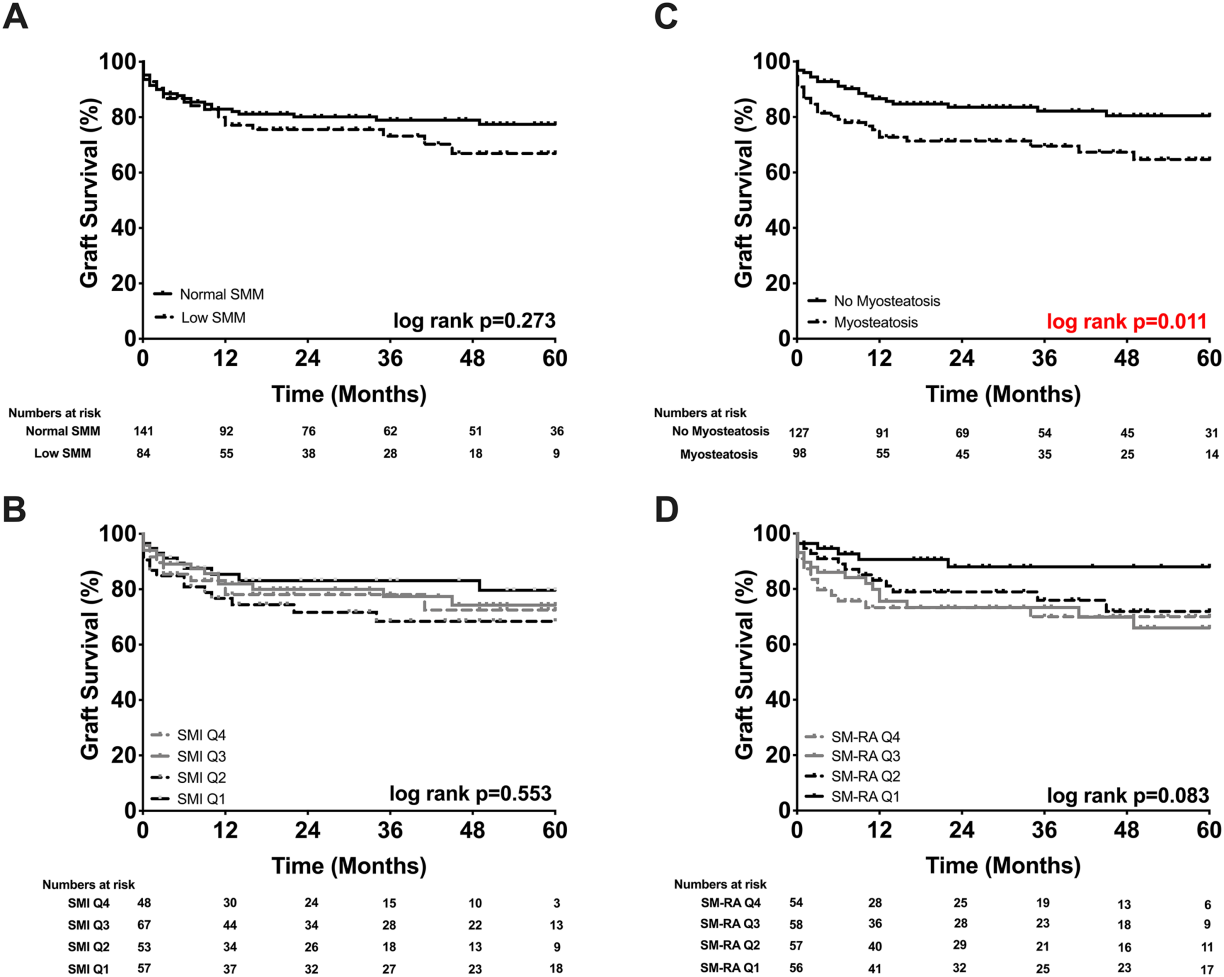
Probability of graft survival stratified by body composition. (A) Normal skeletal muscle mass (SMM) 78% vs. low SMM 67%. (B) Skeletal muscle index (SMI) Q4 73% vs. Q3 75% vs. Q2 68% vs. Q1 80%. (C) No myosteatosis 81% vs. myosteatosis 65%. (D) Skeletal muscle radiation attenuation (SM‐RA) Q4 70% vs. Q3 66% vs. Q2 72% vs. Q1 88%.

There were 19 patients (8%) who died within the first 90 days following OLT. A total of 59 patients died over the follow‐up period (May 2010 to May 2020; see *Table* 3). The probability of graft survival at 5 years was significantly worse for patients with myosteatosis compared with patients with higher muscle density (65% vs. 81%: *P* = 0.011, *Figure* 3). When patients were divided into quartiles based on the SM‐RA values, no significant difference was found in terms of graft survival (SM‐RA Q4 70% vs. Q3 66% vs. Q2 72% vs. Q1 88%; *P* = 0.083, *Figure* 3).

**Figure 4:**
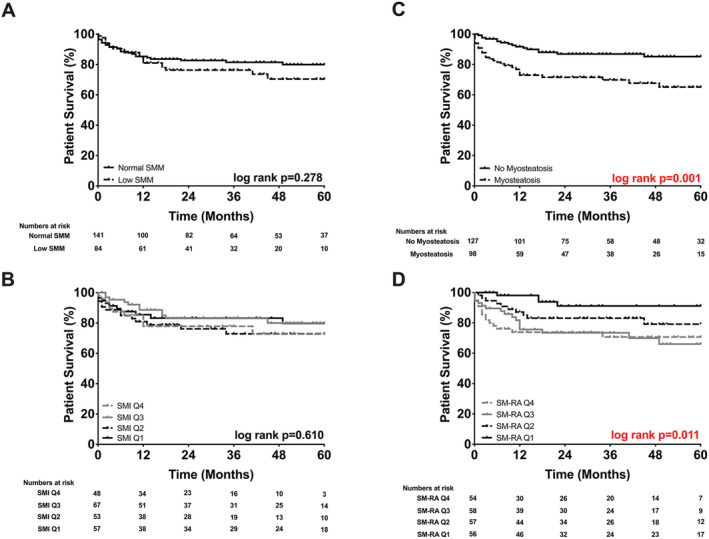
Probability of patient survival stratified by body composition. (A) Normal skeletal muscle mass (SMM) 80% vs. low SMM 70%. (B) Skeletal muscle index (SMI) Q4 73% vs. Q3 80% vs. Q2 73% vs. Q1 80%. (C) No myosteatosis 85% vs. myosteatosis 65%. (D) Skeletal muscle radiation attenuation (SM‐RA) Q4 71% vs. Q3 66% vs. Q2 80% vs. Q1 91%.

Similar to the graft survival rates, the probability of patient survival at 5 years was significantly worse for patients with myosteatosis compared with patients above the defined cut‐offs of SM‐RA (65% vs. 85%: *P* = 0.001, *Figure* 4). When the SM‐RA quartiles were considered, there was also a significant difference in patient survival (SM‐RA Q4 71% vs. Q3 66% vs. Q2 80% vs. Q1 91%; *P* = 0.011, *Figure* 4).

Alterations of muscle mass alone had no significant effects on graft and patient survival, neither as a single cut‐off for low SMM nor as SMI quartiles (*Figures* 3 and 4). Probability of graft and patient survival for individuals with low SMM was 67% and 70% vs. 78% and 80% for individuals without low SMM (*P* = 0.273 and *P* = 0.278) (*Figures* 3 and 4).

**Figure 5:**
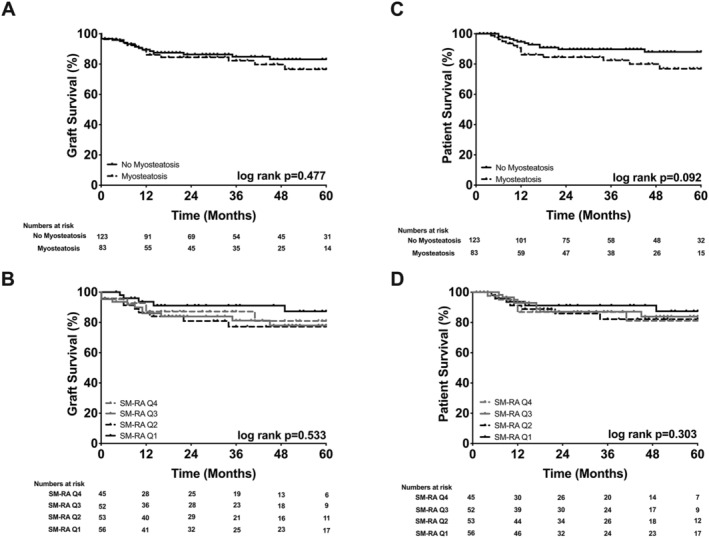
Probability of graft and patient survival stratified by myosteatosis after excluding 90 day mortality. To show the long‐term effects of myosteatosis, subgroup survival analyses were repeated, and patients who died within 90 days of transplantation (*n* = 19) were excluded. (A) Graft survival by no myosteatosis 83% vs. myosteatosis 77%. (B) Graft survival by skeletal muscle radiation attenuation (SM‐RA) Q4 81% vs. Q3 78% vs. Q2 77% vs. Q1 87%. (C) Patient survival by no myosteatosis 88% vs. myosteatosis 77%. (D) Patient survival by SM‐RA Q4 81% vs. Q3 84% vs. Q2 82% vs. Q1 88%.

Because most of the difference in survival occurred during the early post‐OLT phase (*Figures* 3 and 4), emphasizing the significant effects of myosteatosis on perioperative outcomes,^14^ secondary survival analyses with exclusion of patients who have died within the first 90 days after OLT (*n* = 19) were carried out (*Figure* 5). Interestingly, myosteatosis lost its prognostic value for graft and patient survival (*P* = 0.011 vs. *P* = 0.477 and *P* = 0.001 vs. *P* = 0.092, respectively; see *Figures* 3, 4, 5). The significant difference of the log‐rank test between the SM‐RA quartiles for patient survival was also lost when individuals with early mortality were excluded from the analysis (*P* = 0.011 vs. *P* = 0.303, *Figures* 4 and 5).

We next performed univariable and multivariable Cox regression analyses to identify independent risk factors for graft loss and overall mortality. Although our univariable Cox proportional hazards regression model showed a relevant association of recipient pre‐transplant ICU stay [odds ratio (OR) 2.213, 95% confidence interval (CI) 1.256–3.899, *P* = 0.006], KPS (OR 1.773, 95% CI 1.018–3.088, *P* = 0.043), intraoperative transfusion of red blood cell units (OR 2.252, 95% CI 1.232–4.116, *P* = 0.008), and the presence of myosteatosis (OR 2.025, 95% CI 1.154–3.553, *P* = 0.014) with graft survival, none of these factors remained significant in the final multivariable model (Supporting Information, *Tables* S1 and 2).

**Table 2:** Multivariable Cox regression analysis of prognostic factors for graft and patient survival

	Graft survival—multivariable analysis		Patient survival—multivariable analysis	
	Hazard ratio (95% confidence interval)	*P*‐value	Hazard ratio (95% confidence interval)	*P*‐value
Pre‐transplant labMELD ≥ 25	n.a.	n.a.	0.767 (0.318–1.850)	0.555
Recipient pre‐transplant ICU yes	1.969 (0.780–4.967)	0.151	2.169 (0.854–5.510)	0.104
Karnofsky performance score <60	0.801 (0.317–2.020)	0.638	1.197 (0.339–3.597)	0.748
Intraoperative RBC units ≥15	1.735 (0.908–3.316)	0.095	1.446 (0.732–2.855)	0.288
Myosteatosis (SM‐RA) yes	1.716 (0.937–3.143)	0.080*	2.260 (1.177–4.340)	0.014^#^

ICU, intensive care unit; MELD, model for end‐stage liver disease; RBC, red blood cell; SM‐RA, skeletal muscle radiation attenuation.

Results from the Cox proportional hazards regression model were given as hazard ratios (HR) with 95% confidence intervals. Factors showing significant results in the univariable analysis (see *Table* S1) were included into the multivariable logistic regression model. Only significant results are shown. To avoid a multicollinearity effect, certain variables were not included into the Cox regression model.

* When removing patients with perioperative mortality (90 day mortality, *n* = 19), myosteatosis loses its significant effect on graft survival (hazard ratio: 1.290, 95% confidence interval: 0.636–2.617, *P* = 0.481 in univariable analysis).

^#^ When removing patients with perioperative mortality, myosteatosis loses its significant effect on patient survival (hazard ratio: 1.914, 95% confidence interval: 0.885–4.141, *P* = 0.099 in univariable analysis).

The multivariable analysis has identified myosteatosis as an independent factor associated with impaired patient survival (OR 2.260, 95% CI 1.177–4.340, *P* = 0.014, *Tables* S1 and 2). In contrast, although labMELD (OR 1.909, 95% CI 1.060–3.439, *P* = 0.031), pre‐transplant ICU stay (OR 2.798, 95% CI 1.553–4.041, *P* = 0.001), KPS (OR 2.399, 95% CI 1.327–4.336, *P* = 0.004), and intraoperative transfusion of red blood cell units (OR 2.102, 95% CI 1.109–3.987, *P* = 0.023) showed significant hazard ratios (HRs) in the univariable analysis, they lost their significance in the multivariable model (*Tables* S1 and 2).

In line with the observations made in the Kaplan–Meier analysis and log‐rank tests, the significant association of myosteatosis with a decreased graft and patient survival was lost already in the univariable analysis when early mortality (90 day mortality, *n* = 19) was excluded from the analysis resulting in an HR of 1.290 (0.636–2.617) and a *P*‐value = 0.481 for graft survival and an HR of 1.914 (0.885–4.141) and a *P*‐value = 0.099 for patient survival (*Tables* S1 and 2).

Furthermore, patients with myosteatosis showed significantly higher all‐cause mortality (37% vs. 18%: *P* = 0.002) and a higher incidence of deaths due to respiratory and septic complications (8% vs. 2%: *P* = 0.022 and 16% vs. 8%: *P* = 0.049, respectively; see *Table* 3).

**Table 3:** Cause of death for all patients died until the time point of last follow‐up

Cause of death	All patients (*n* = 225)	Myosteato sis (*n* = 98)	No myosteatosis (*n* = 127)	*P*‐value
All	59 (26)	36 (37)	23 (18)	0.002
Primary non‐function	2 (1)	2 (2)	0 (0)	0.189
Gastrointestinal	1 (<1)	1 (1)	0 (0)	0.436
Respiratory	10 (4)	8 (8)	2 (2)	0.022
Recurrent liver disease	7 (3)	3 (3)	4 (3)	0.970
Septic	26 (12)	16 (16)	10 (8)	0.049
Malignancy	6 (3)	2 (2)	4 (3)	0.699
Other	7 (3)	4 (4)	3 (2)	0.408

Total follow‐up period reported: May 2010 to May 2020.

## Discussion

This study explores the association of myosteatosis with long‐term post‐OLT graft and patient survival. While there was a high incidence of pathological BC alterations in our cohort with more than 40% of our patients suffering from myosteatosis and 37% presenting with low SMM, we here show a limited long‐term prognostic role of myosteatosis and low SMM in deceased donor OLT. Interestingly, based on our data, the prognostic value of myosteatosis seems to be accentuated in the early post‐operative phase, as BC loses its prognostic value on long‐term outcomes when patients with early mortality are excluded.

Because the gap between allograft supply and demand continues to increase, the optimal risk stratification and utilization of the available donor pool are based not only on conventional risk factors but also on nutritional donor–recipient characteristics.^13^, ^14^, ^31^ While a handful of reports have suggested a potential role of myosteatosis in clinical outcome following OLT, the majority of previous studies focused on sarcopenia and on Asian cohorts of living donor liver transplantation (LDLT).^1^, ^6^, ^9^, ^14^, ^32^, ^33^ In addition, recent data from our group and others suggest that myosteatosis, as characterized by the presence of intermyocellular and intramyocellular fat depositions, may result in dysregulated pathophysiological responses and consequential inferior clinical outcomes even in patients with normal or slightly reduced muscle mass.^6^, ^14^ In a recent report by Hamaguchi *et al*., the authors investigated CT‐based BC in a single‐centre Japanese cohort of 657 living liver donors and identified SMI and intramuscular adipose tissue content (IMAC) as independent predictors of post‐transplant recipient survival.^33^ A further study by the same group evaluated IMAC and psoas muscle mass index in 200 adult recipients undergoing LDLT and demonstrated a significant association of recipient mortality with high IMAC and low psoas muscle mass index.^32^ Furthermore, Bhanji *et al*. reported a correlation of myosteatosis with hepatic encephalopathy and waiting list mortality in a large cohort of 675 cirrhotic patients.^3^ Although, myosteatosis was previously linked to inferior waiting list survival in end‐stage liver disease and in patients undergoing LDLT, its specific effects on graft and patient survival in patients undergoing deceased donor OLT remained to be determined.^1^, ^3^, ^9^, ^14^, ^34^

Recently, our group investigated the role of myosteatosis and low SMM in early perioperative outcomes.^14^ Patients with myosteatosis experienced a higher number and more severe surgical complications over the first 3 months following OLT. Also, increased rates of early allograft dysfunction, higher comprehensive complication index scores, longer ICU and hospital stays, higher procedural costs, and an increased need for intraoperative blood transfusions were seen in patients with myosteatosis.^14^ Of note, low SMM alone was not associated with any of the previously perioperative outcome parameters. To further explore this observation, we investigated the prognostic role of muscle quality (myosteatosis) and low SMM in post‐OLT graft and patient survival. Patients were divided into low and normal SM‐RA and SMI cohorts and were also stratified into SM‐RA and SMI quartiles (*Figures* 2, 3, 4, 5). For this, we used validated cut‐off values from large cohorts of patients with chronic liver disease, adopting recent recommendations of the North American expert group on fitness, life enhancement, and exercise in liver transplantation.^6^, ^8^, ^19^, ^35^

In the present study, the probability of graft and patient survival at 5 years was significantly worse in the presence of myosteatosis. Our quartile‐based analysis of patient survival also showed significant differences between the various SM‐RA quartiles. Patients belonging to the first SM‐RA quartile (thus having the highest muscle density) demonstrated an excellent 91% patient survival compared with 66% and 71% in Q3 and Q4, respectively (*P* = 0.011).

Subsequently, BC parameters were fitted into a Cox proportional hazards regression model to further assess the association of low SMM and myosteatosis with graft and patient survival in our cohort. Here, we identified the presence of myosteatosis as an independent predictor of inferior 5 year patient survival in the final multivariable model. Interestingly, the significant effect of myosteatosis on graft and patient survival was lost in our secondary univariable and multivariable analyses where patients who died within the first 90 days after OLT were excluded. Low SMM did not show any significant hazard ratios in the analysis for graft and patient survival.

Next, cause of mortality over the observation period was analysed in detail to further explore the role myosteatosis in mortality. Patients with myosteatosis showed not only significantly higher all‐cause mortality, but also death due to respiratory and septic complication were more frequent in the myosteatosis subcohort. This is in line with previous findings, showing that structural alteration of the skeletal muscle compartment and muscle wasting are associated with infectious and respiratory complications, not only in liver disease and OLT but also in various oncological entities.^1^, ^16^, ^36^, ^37^

Although myosteatosis can occur when lipid intake simply exceeds the disposal capacity of the human body,^7^, ^14^, ^38^ pathological fat deposition was also confirmed in non‐obese or even in underweight patients,^14^, ^39^ highlighting that mechanisms other than exogenous lipid intake (e.g. liver–muscle crosstalk and alterations of lipoprotein metabolism in liver disease) may play an important role in the development of myosteatosis in patients with chronic liver disease.^6^, ^7^, ^14^ These observations support previous findings on the potent short‐term effects of myosteatosis and low muscle density.^6^, ^14^ While inflammatory responses play a pivotal role in early ischaemia–reperfusion injury of the liver allograft (ischaemic complications and rejection),^40^ a pro‐inflammatory tissue micro‐environment as it is the case in the presence of myosteatosis likely also impacts early graft and patient survival following OLT.

The findings of this study should be interpreted in the light of potential limitations. First, due to the inherent uncontrolled, retrospective, and single‐centre nature of our analysis, no preoperative functional assessment of fitness, muscle strength, and nutritional status was possible.^9^, ^14^ Second, despite our observation and important conclusion on the effects of myosteatosis on clinical outcome (predominantly short‐term), there was still a non‐significant difference in the Kaplan–Meier curves for graft and patient survival even after exclusion of early mortality (*Figure* 4). It is therefore reasonable to assume that our analysis may have also been limited by the sample size and a relatively heterogeneous study population.

Notwithstanding the aforementioned limitations, we identified recipient myosteatosis as an important prognostic marker for clinical outcomes following deceased donor OLT. The prognostic value of myosteatosis seems to be particularly important in the early post‐OLT phase. This observation is not only important for our understanding on how inter‐individual alterations of BC influence clinical outcomes in these patients, but it may also represent an important therapeutic target for interventions during the perioperative phase. Validation in prospective interventional clinical trials is warranted.

## Funding

This research project was in part supported by the START Program (136/17 to G.L. and 23/19 to Z.C.) and the Clinician Scientist Program (to Z.C.) of the Faculty of Medicine, RWTH Aachen University and by the Excellence Initiative of the German federal and state governments (G:(DE‐82) ZUK2‐SF‐OPSF443 to G.L.) without involvement of the funders in study design, data collection, data analysis, manuscript preparation, or decision to publish.

## Author contributions

The study was designed by the initiating study team (Z.C., G.L., and U.P.N.). Data collection and analysis were performed by Z.C., W.K., I.L., J.B., H.M., P.S., S.A.L., T.F.U., P.B., U.P.N., and G.L. Manuscript was drafted by Z.C., G.L., M.W.v.W., and W.K. Further authors (W.K., I.L., H.M., J.B., S.A.L., T.F.U., P.B., P.S., C.T., M.W.v.W., F.T., U.P.N., and G.L.) have substantially contributed to the final version of the manuscript. All authors have read and approved the final version of the manuscript.

## Conflict of interest

None declared.

## Supporting information


**Table S1.** Univariable Cox regression analysis for graft‐ and patient survival
