# Supplementary material for: The role of recipient myosteatosis in graft and patient survival after deceased donor liver transplantation
Source: J Cachexia Sarcopenia Muscle. 2021 Feb 1;12(2):358–67. doi: 10.1002/jcsm.12669 (PMC8061365; doi:10.1002/jcsm.12669)
Supplement: Supplementary file 1 — Table S1. Univariable Cox regression analysis for graft‐ and patient survival [file JCSM-12-358-s001.pdf]

Supplementary Table 1. Univariable Cox regression analysis for graft- and patient survival

		**Graft survival - Univariable analysis**		**Patient survival – Univariable analysis**	
	**n (%)**	**Hazard ratio (95% Confidence Interval)**	**p value**	**Hazard ratio (95% Confidence Interval)**	**p value**
**Donor age** ≥60 years	94 (42)	1.375 (0.789-2.496)	0.261	1.396 (0.778-2.504)	0.264
**Donor BMI** ≥25	178 (79)	1.623 (0.730-3.608)	0.235	1.448 (0.646-3.242)	0.368
**Donor Sex** Male	117 (52)	1.040 (0.597-1.812)	0.890	1.120 (0.623-2.012)	0.705
**Pre-transplant Child-Pugh Score** ≥7	139 (62)	1.333 (0.736-2.416)	0.343	1.792 (0.925-3.471)	0.084
**ECD** Yes	154 (68)	1.723 (0.882-3.365)	0.111	1.719 (0.851-3.472)	0.131
**Recipient age** ≥60 years	87 (39)	1.175 (0.670-2.060)	0.574	1.461 (0.813-2.625)	0.205
**Recipient BMI** ≥25	154 (68)	0.991 (0.541-1.816)	0.977	0.962 (0.512-1.809)	0.905
**Recipient Sex** Male	150 (67)	0.826 (0.464-1.472)	0.517	0.699 (0.385-1.270)	0.240
**Etiology of liver disease**<br>ALF<br>HCC<br>Alcoholic cirrhosis<br>Viral<br>PSC/PBC<br>Graft failure<br>Other	 31 (14)<br>63 (28)<br>45 (20)<br>15 (7)<br>21 (9)<br>4 (2)<br>46 (20)	 0.774 (0.226-2.645)<br>1.604 (0.660-3.901)<br>2.027 (0.818-5.022)<br>1.137 (0.294-4.403)<br>0.900 (0.233-3.484)<br>4.120 (0.506-33.576)<br>1	0.290	 1.134 (0.304-4.225)<br>2.111 (0.676-5.811)<br>2.674 (0.953-7.504)<br>1.704 (0.407-7.152)<br>0.843 (0.163-4.349)<br>1.363 (0.894-36.692)<br>1	0.146
**Pre-transplant labMELD** ≥25	73 (32)	1.490 (0.846-2.626)	0.167	**1.909 (1.060-3.439)**	**0.031**
**Recipient pre-transplant ICU** Yes	56 (25)	**2.213 (1.256-3.899)**	**0.006**	**2.798 (1.553-4.041)**	**0.001**
**Recipient pre-transplant abdominal surgery** Yes	82 (36)	0.779 (0.425-1.427)	0.419	0.926 (0.498-1.722)	0.809
**Recipient pre-transplant encephalopathy** Yes	90 (40)	0.966 (0.548-1.702)	0.905	1.178 (0.654-2.121)	0.586
**Karnofsky Performance Score** <60	87 (39)	**1.773 (1.018-3.088)**	**0.043**	**2.399 (1.327-4.336)**	**0.004**
**Cold ischemic time** ≥480 (min)	126 (56)	0.676 (0.383-1.193)	0.176	0.787 (0.432-1.431)	0.432
**Warm ischemic time** ≥45 min	116 (52)	0.983 (0.558-1.734)	0.954	1.070 (0.588-1.948)	0.825
**Intraoperative RBC Units** ≥15	36 (16)	**2.252 (1.232-4.116)**	**0.008**	**2.102 (1.109-3.987)**	**0.023**
**Low SMM (SMI)** Yes	84 (37)	1.361 (0.778-2.381)	0.280	1.381 (0.766-2.488)	0.283
**Myosteatosis (SM-RA)** Yes	98 (44)	**2.025 (1.154-3.553)**	**0.014**	**2.758 (1.497-5.082)**	**0.001**

Values were given as numbers and (per cent). Results from the Cox proportional hazards regression model were given as hazard ratios (HR) with 95% confidence intervals (95% CI). Factors showing significant results in the univariable analysis were included into the multivariable logistic regression model (see main manuscript). Only significant results are shown. To avoid a multicollinearity effect, certain variables were not included into the Cox regression model.

Abbreviations used: BMI: body mass index, ECD: extended criteria donor allografts, ALF: acute liver failure, HCC: hepatocellular carcinoma, PSC: primary sclerosing cholangitis, PBC: primary biliary cholangitis, MELD: model for end-stage liver disease, RBC: red blood cell units, SMM: skeletal muscle mass; SMI: lumbar 3 skeletal muscle index, SM-RA: lumbar 3 skeletal muscle radiation attenuation
